# Polyphenol-Rich Fraction of *Ecklonia cava* Improves Nonalcoholic Fatty Liver Disease in High Fat Diet-Fed Mice

**DOI:** 10.3390/md13116866

**Published:** 2015-11-12

**Authors:** Eun-Young Park, Hojung Choi, Ji-Young Yoon, In-Young Lee, Youngwan Seo, Hong-Seop Moon, Jong-Hee Hwang, Hee-Sook Jun

**Affiliations:** 1 College of Pharmacy, Mokpo National University, Muan-gun, Jeonnam 58554, Korea; E-Mails: parkey@mokpo.ac.kr (E.-Y.P.); hbsmoon@mokpo.ac.kr (H.-S.M.); 2 College of Pharmacy, Gachon Institute of Pharmaceutical Science, Gachon University, Yeonsu-gu, Incheon 21999, Korea; E-Mails: hchoi@gachon.ac.kr (H.C.); yoonj6@naver.com (J.-Y.Y.); 3 Korea Mouse Metabolic Phenotyping Center (KMMPC), Lee Gil Ya Cancer and Diabetes Institute, Gachon University, Yeonsu-gu, Incheon 21999, Korea; E-Mail: 76pey@naver.com; 4 Ocean Science & Technology School, Korea Maritime and Ocean University, Yeongdo-gu, Busan 49112, Korea; E-Mail: ywseo@kmou.ac.kr; 5 Gachon Medical Research Institute, Gil Hospital, Namdong-gu, Incheon 21565, Korea

**Keywords:** *Ecklonia cava*, hepatic steatosis, MRI, MRS, fatty liver, NAFLD

## Abstract

*Ecklonia cava* (*E. cava*; CA) is an edible brown alga with beneficial effects in diabetes via regulation of various metabolic processes such as lipogenesis, lipolysis, inflammation, and the antioxidant defense system in liver and adipose tissue. We investigated the effect of the polyphenol-rich fraction of *E. cava* produced from Gijang (G-CA) on nonalcoholic fatty liver disease (NAFLD) in high-fat diet (HFD)-fed mice. C57BL6 mice were fed a HFD for six weeks and then the HFD group was administered 300 mg/kg of G-CA extracts by oral intubation for 10 weeks. Body weight, fat mass, and serum biochemical parameters were reduced by G-CA extract treatment. MRI/MRS analysis showed that liver fat and liver volume in HFD-induced obese mice were reduced by G-CA extract treatment. Further, we analyzed hepatic gene expression related to inflammation and lipid metabolism. The mRNA expression levels of inflammatory cytokines and hepatic lipogenesis-related genes were decreased in G-CA-treated HFD mice. The mRNA expression levels of cholesterol 7 alpha-hydroxylase 1 (CYP7A1), the key enzyme in bile acid synthesis, were dramatically increased by G-CA treatment in HFD mice. We suggest that G-CA treatment ameliorated hepatic steatosis by inhibiting inflammation and improving lipid metabolism.

## 1. Introduction

Nonalcoholic fatty liver disease (NAFLD), a hepatic manifestation of metabolic syndrome, is the most common hepatic pathology and its incidence is increasing rapidly worldwide [1,2]. NAFLD is characterized by abnormal lipid deposition in the liver without alcohol consumption and is caused by insulin resistance, obesity, and metabolic disorders [3,4,5]. The term NAFLD covers simple steatosis and non-alcoholic steatohepatitis (NASH), a condition wherein steatosis is accompanied with inflammation and fibrosis [6]. Some studies suggest that NAFLD puts patients at an increased risk for atherosclerosis and cardiovascular disease [7,8,9,10]. Insulin sensitizers, such as metformin and thiazolidinediones, and hepatoprotective agents have shown therapeutic effects; however, there is no approved pharmacological remedy for NAFLD [11].

*Ecklonia cava* (*E.cava*; CA), a brown alga, is found abundantly in the subtidal regions of Korea and used as a foodstuff in Korea and Japan [12]. CA contains polyphenolic compounds, phlorotannins, which have diverse biological activities including anti-inflammatory, anti-oxidative, anti-cancer, and anti-diabetic effects [13,14]. CA extract improves insulin resistance, glucose tolerance, and lipid metabolism in the high-fat diet (HFD)-induced obesity model [15,16]. With regard to its improvement of liver function, CA polyphenol suppresses hepatic stellate cell activation due to high glucose levels and protects against ethanol-induced liver fibrosis through the inhibition of reactive oxygen species [17,18].

We have previously demonstrated that treatment with polyphenol-enriched G-CA (CA produced from Gijang) extract reduced weight gain and blood glucose levels owing to its anti-inflammatory effects and improvement of lipid metabolism in HFD-fed mice [19]. In the present study, we focused on the therapeutic effect of CA on obesity-induced metabolic disease, while the previous paper aimed to investigate aspects of the preventive effect of CA. We evaluated the efficacy of G-CA extract against fatty liver in diet-induced obese mice using magnetic resonance imaging (MRI) and magnetic resonance spectroscopy (MRS) *in vivo* imaging techniques to assess liver fat and liver volume quantitatively. In addition, we analyzed lipid levels in serum and the liver, and hepatic gene expression related to inflammation and lipid metabolism.

## 2. Results

### 2.1. Reduction of Adiposity in G-CA-HFD Mice

**Figure 1:**
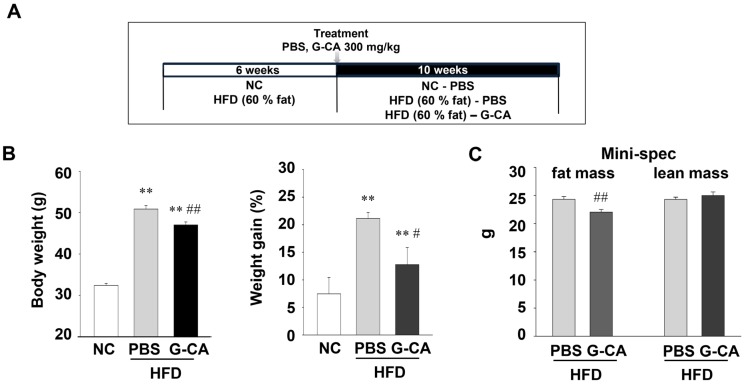

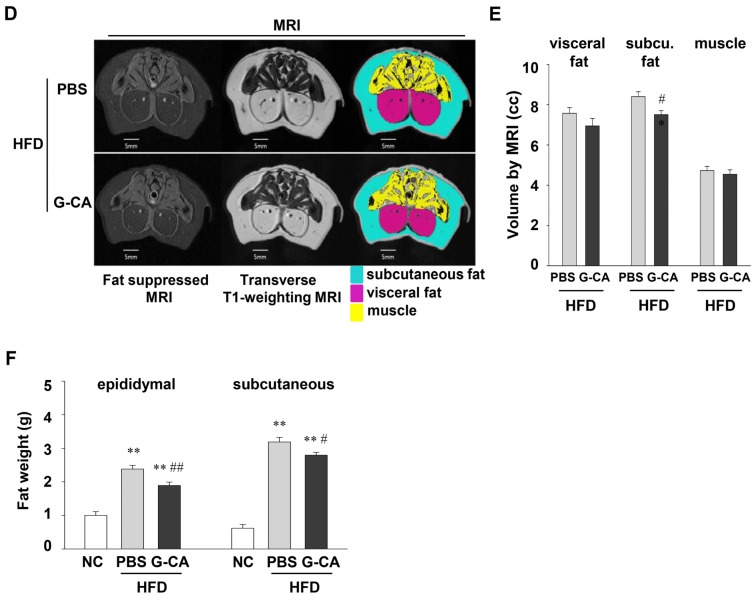
Effect of G-CA (*E. cava* produced from Gijang) on body weight and fat content. Six weeks after beginning a high fat diet, C57BL6 mice were orally administered G-CA (300 mg/kg body weight) or PBS daily: (**A**) A diagram of experimental procedure. (**B**) After 10 weeks of G-CA treatment, body weights and weight gain were measured (NC: *n* = 5, PBS-HFD: *n* = 9, G-CA-HFD: *n* = 7). (**C**) Whole body fat and lean mass were measured by a ^1^H minispec system. (**D**) From the left to the right, 1D shows fat-suppressed, T1-weighted, and segmented MRI of the same transverse slice of a mouse (*n* = 5). The upper and lower rows show the images from the PBS-HFD and the G-CA-HFD groups, respectively. Segmented images on the right depict segmented regions in 3 different colors (blue: subcutaneous fat, pink: visceral fat, yellow: muscle). (**E**) Volumes of various tissues (visceral fat, subcutaneous fat, and total muscle) were quantitated from MRI data. (**F**) Epididymal fat and subcutaneous fat pad were collected and weighed. NC: untreated, normal chow diet; PBS-HFD: PBS-treated, high fat diet (HFD); and G-CA-HFD: G-CA-treated, HFD. Values are mean ± SE. ** *p* < 0.01 *vs.* NC group; ^#^ *p* < 0.05, ^##^ *p* < 0.01 *vs.* PBS-HFD group.

Since NAFLD pathogenesis is related to obesity, measuring weight loss is a reasonable approach for evaluating NAFLD treatment [20,21]. Therefore, we measured whether CA treatment can reduce the body weight gain in HFD-fed mice. CA-HFD mice had low body weights and less weight gain (39.64% decrease) compared with the PBS-HFD mice (Figure 1B and Figure S1). The amount of food consumption per day over the 10-week period was not significantly different between PBS-HFD and G-CA-HFD (Figure S1). To investigate whether G-CA treatment affects body composition, we measured fat and lean mass using NMR, non-invasive body composition analyzer. Total fat mass was significantly decreased in the G-CA-treated group compared with that PBS-treated group, whereas lean mass was not different between the two groups (Figure 1C). The regional fat and skeletal muscle volumes were analyzed by MRI. As shown in Figure 1D,E, in the G-CA-HFD mice group visceral and whole body subcutaneous fat were reduced as compared to the PBS-HFD mice group (visceral fat: 7.6 ± 0.6 *vs.* 7.0 ± 0.9 mL; whole body subcutaneous fat 8.3 ± 0.5 *vs.* 7.5 ± 0.5 mL, *p* < 0.05). In addition, little difference was found in response to G-CA treatment in muscle volume of the whole body. The subcutaneous and epididymal fat pad weights of G-CA-HFD mice were significantly decreased compared with the PBS-HFD mice (Figure 1F).

### 2.2. Reduced Liver Volume in G-CA-HFD Mice

**Figure 2:**
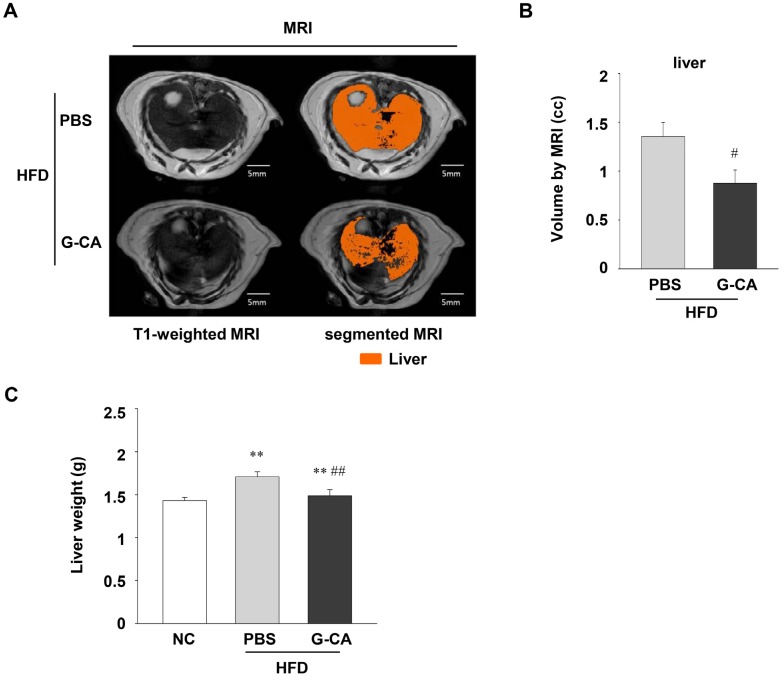
Effect of G-CA on liver volume and liver tissue weight. Six weeks after beginning a high fat diet, C57BL6 mice were orally administered G-CA (300 mg/kg body weight) or PBS daily. (**A**) After 10 weeks of G-CA treatment, figure shows typical MRIs of liver of PBS-HFD (*n* = 5) and G-CA-HFD groups (*n* = 5). The left panels show T1-weighted MRI including the liver. The sections in orange-brown represent liver in the segmented MRIs. (**B**) The graph depicts the difference in the total liver volumes in PBS-HFD and G-CA-HFD groups. (**C**) After 10 weeks of G-CA treatment, liver tissue was collected and weighed (NC: *n* = 5, PBS-HFD: *n* = 9, G-CA-HFD: *n* = 7). NC: untreated, normal chow diet; PBS-HFD: PBS-treated, high fat diet (HFD); and G-CA-HFD: G-CA-treated, HFD. Values are mean ± SE. ** *p* < 0.01 *vs.* NC group; ^#^ *p* < 0.05, ^##^ *p* < 0.01 *vs.* PBS-HFD group.

Numerous studies had reported increased liver weight in HFD-induced NAFLD as a consequence of hepatic lipid accumulation [22,23]. To examine whether treatment with G-CA extract affects liver content in HFD-induced NAFLD, we measured the volume of liver using MRI (Figure 2A). As shown in Figure 2B, liver volume significantly decreased in the G-CA-HFD group compared with that of the PBS-HFD group (35.56% decrease). When we measured liver weight after sacrifice, the increase in liver weight observed in the HFD groups was significantly attenuated by G-CA treatment (Figure 2C).

### 2.3. Reduction of Intrahepatic Lipid Accumulation in G-CA-HFD Mice

**Figure 3:**
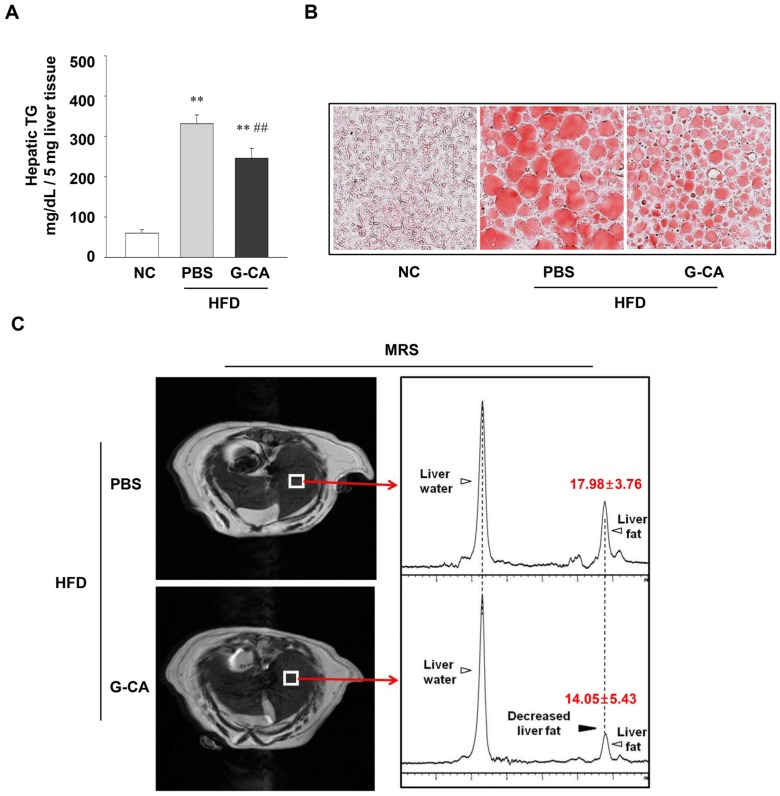
Effect of G-CA on hepatic lipid accumulation. Six weeks after beginning a high fat diet, C57BL6 mice were orally administered G-CA (300 mg/kg body weight) or PBS daily for 10 weeks. (**A**) Hepatic triglyceride (TG) content was measured in liver tissue (*n* = 5–6); (**B**) Oil Red O staining was performed on frozen liver sections; (**C**) The right panels show typical liver ^1^H MR spectra from the selected region denoted as the white square in MRI in the left in PBS-HFD (*n* = 5) and G-CA-HFD groups (*n* = 5). NC: untreated, normal chow diet; PBS-HFD: PBS-treated, high fat diet (HFD); and G-CA-HFD: G-CA-treated, HFD. Values are mean ± SE. ** *p* <0.01 *vs.* NC group; ^##^ *p* <0.01 *vs.* PBS-HFD group.

To examine whether G-CA treatment can ameliorate hepatic steatosis, we measured TG levels in HFD-fed mice. The HFD feeding increased hepatic TG levels by about 550% (Figure 3A). The HFD-induced increases in hepatic TG levels were significantly decreased by G-CA treatment (Figure 3A; about 26% decrease). Measurement of hepatic lipid contents in frozen section showed that lipid droplet accumulation reduced in G-CA-HFD mice compared with that in PBS-HFD mice (Figure 3B). MRS measurements were also performed to quantify hepatic lipid content; the results showed that the fat contents of the liver were reduced by about 22% in G-CA-HFD mice compared with PBS-HFD mice (Figure 3C).

### 2.4. Improvement of Liver Injury and Serum Lipid Levels in G-CA-HFD Mice

**Figure 4:**
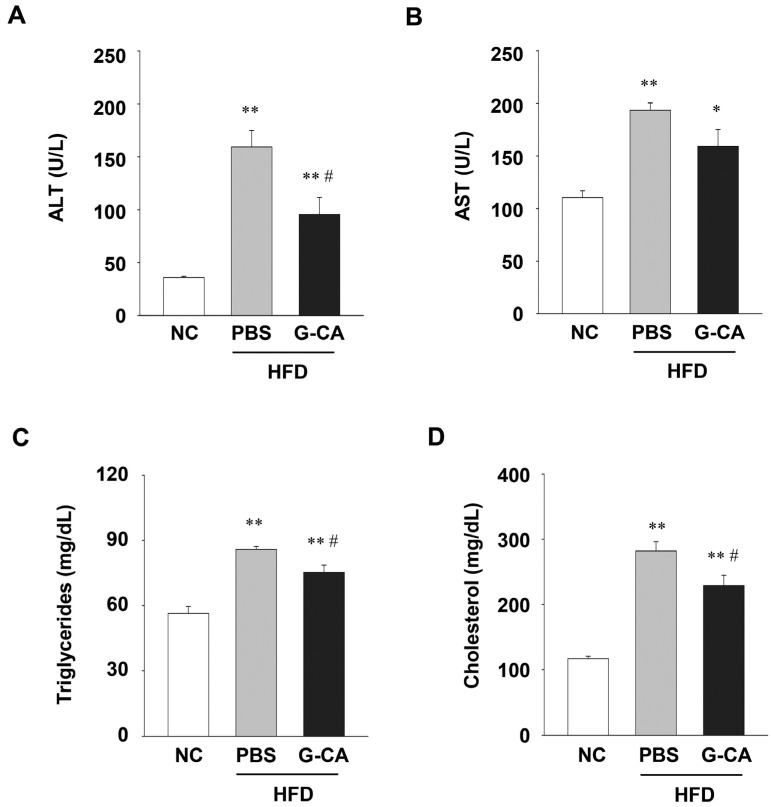

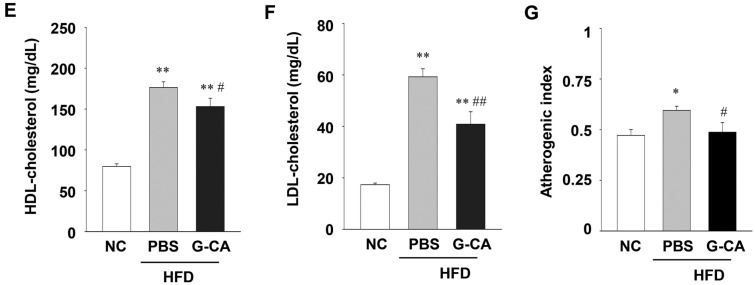
Effect of G-CA on serum biochemical parameters. Six weeks after beginning a high fat diet, C57BL6 mice were orally administered G-CA (300 mg/kg body weight) or PBS daily. After 10 weeks, blood samples were collected and measured biochemical parameters (*n* = 5–6): (**A**) alanine aminotransferase (ALT); (**B**) aspartate aminotransferase (AST); (**C**) triglycerides; (**D**) cholesterol; (**E**) high density lipoprotein (HDL)-cholesterol; (**F**) low density lipoprotein (LDL)-cholesterol; and (**G**) atherogenic index. NC: untreated, normal chow diet; PBS-HFD: PBS-treated, high fat diet (HFD); and G-CA-HFD: G-CA-treated, HFD. Values are mean ± SE. * *p* < 0.05, ** *p* < 0.01 *vs.* NC group; ^#^ *p* < 0.05, ^##^ *p* < 0.01 *vs.* PBS-HFD group.

Excessive fat accumulation in the liver causes hepatocellular injury [24]. To evaluate whether G-CA treatment improves liver injury, serum ALT and AST levels were measured. Serum ALT and AST levels in the HFD-fed mice were significantly increased compared with NC mice, (444% and 175%, respectively; Figure 4A,B). Both ALT and AST levels were decreased in G-CA-HFD mice, but only serum ALT levels were statistically significant (Figure 4A,B; about 40.2% decrease). Next, to determine whether treatment with G-CA extract improves lipid metabolism, we measured the serum TG and cholesterol levels. The serum TG, total cholesterol, HDL-cholesterol and LDL-cholesterol levels were significantly higher in HFD mice compared with NC mice, whereas these serum lipid levels were significantly lower in G-CA-HFD mice compared with PBS-HFD mice (Figure 4C–F). Analysis of the atherogenic index showed that dyslipidemia caused by obesity was also improved by G-CA treatment (Figure 4G). These results suggest that G-CA extract treatment ameliorated liver injury and reduced lipid profiles.

### 2.5. Effects of G-CA Extract on mRNA Expression of Inflammatory Genes, Hepatic Lipogenic Genes and Cholesterol Metabolism-Related Genes

**Figure 5:**
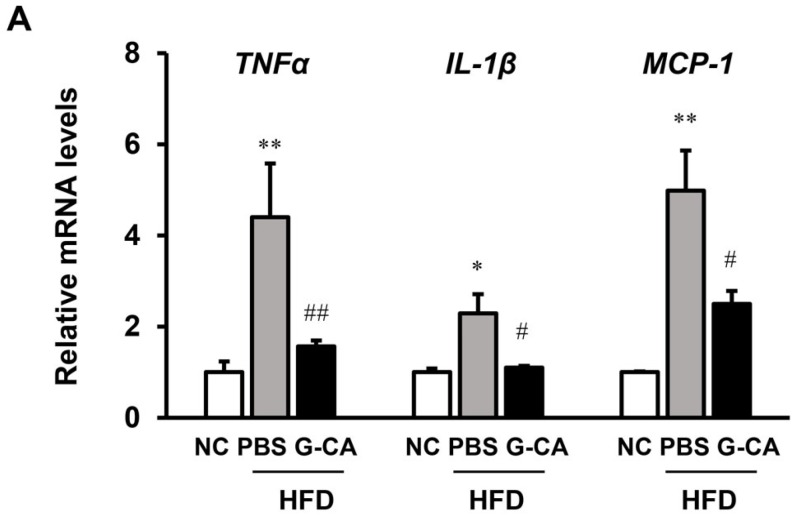

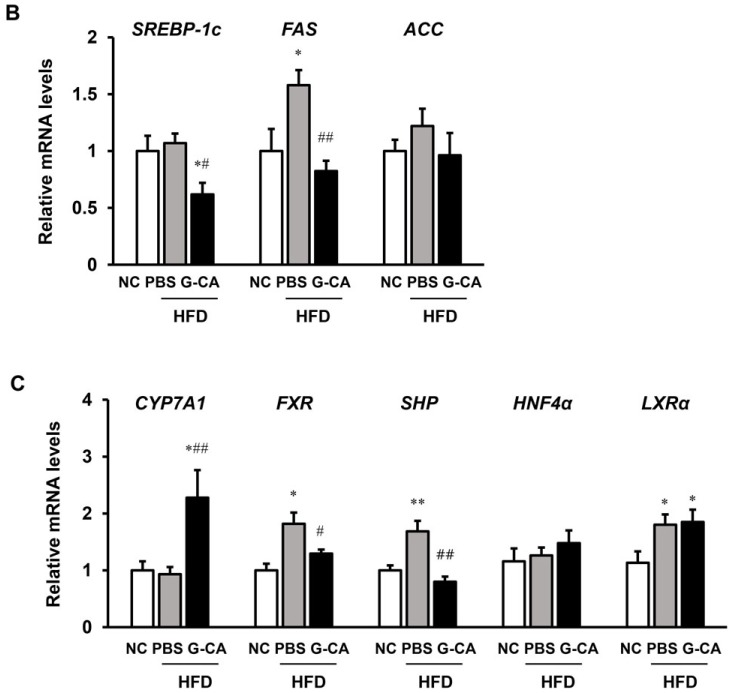
Effect of G-CA on metabolic gene expression in liver. Six weeks after beginning a high fat diet, C57BL6 mice were orally administered G-CA (300 mg/kg body weight) or PBS daily for 10 weeks (*n* = 5–6). (**A**) Tumor necrosis factor alpha (TNFα), interleukin-1 beta (IL-1β), and monocyte chemoattractant protein 1 (MCP-1) mRNA levels were analyzed by RT-qPCR and normalized to 18S rRNA expression; (**B**) Sterol regulatory element-binding protein 1c (SREBP-1c), fatty acid synthase (FAS), and acetyl-CoA carboxylase (ACC) mRNA levels were analyzed by RT-qPCR and normalized to 18S rRNA expression; (**C**) Hepatic cholesterol 7 alpha-hydroxylase 1 (CYP7A1), farnesoid X receptor (FXR), small heterodimer partner (SHP), hepatocyte nuclear factor 4 alpha (HNF4α), and liver X nuclear receptor alpha (LXRα) mRNA levels were analyzed by RT-qPCR and normalized to 18S rRNA expression. Values are expressed as fold change compared with the NC group. NC: untreated, normal chow diet; PBS-HFD: PBS-treated, high fat diet (HFD); G-CA-HFD: G-CA-treated, HFD. Values are mean ± SE. * *p* < 0.05, ** *p* < 0.01 *vs.* NC group; ^#^ *p* < 0.05, ^##^ *p* < 0.01 *vs.* PBS-HFD group.

Inflammation is an important risk factor for the progression of NAFLD [24]. Therefore, we analyzed whether G-CA treatment can improve inflammatory signaling in the liver of HFD-fed mice. The mRNA expression levels of the inflammatory marker genes, tumor necrosis factor alpha (TNF-α), interleukin 1 beta (IL-1β) and monocyte chemoattractant protein 1 (MCP-1) were significantly increased in the PBS-HFD mice as compared with NC mice. TNF-α, IL-1β, and MCP-1 mRNA levels were significantly reduced in the G-CA-HFD group compared with PBS-HFD group (Figure 5A).

To investigate whether G-CA treatment controls hepatic lipogenic gene expression, we analyzed the expression of sterol regulatory element-binding protein 1c (SREBP-1c), fatty acid synthase (FAS), and acetyl-CoA carboxylase (ACC) mRNA. The mRNA levels of SREBP-1c and ACC were increased in PBS-HFD mice compared with NC mice but the difference was not statistically significant. The mRNA expression of FAS was significantly increased in HFD mice compared with PBS-HFD mice (Figure 5B). SREBP-1c and FAS mRNA levels were significantly decreased in G-CA-HFD mice compared with PBS-HFD mice.

Because cholesterol signaling pathway dysfunction increases the risk of NAFLD, cholesterol 7 alpha-hydroxylase 1 (CYP7A1), a key regulator of cholesterol metabolism, is considered to be a potential therapeutic target for the treatment of NAFLD. The mRNA levels of CYP7A1 were significantly increased in the G-CA-HFD mice compared with NC and PBS-HFD mice (Figure 5C). We then examined the mRNA expression of several transcriptional regulators of CYP7A1. The hepatic farnesoid X receptor (FXR)/small heterodimer partner (SHP) pathway is a negative regulator of the expression of *CYP7A1* genes [25]. The mRNA levels of FXR and SHP significantly were decreased in G-CA-HFD mice compared with NC and PBS-HFD mice (Figure 5C). The mRNA levels of liver X nuclear receptor alpha (LXRα) and hepatocyte nuclear factor 4 alpha (HNF4α), both transcriptional regulators of CYP7A1 expression, were not changed by G-CA treatment (Figure 5C). These results suggest that inhibition of the FXR/SHP pathway by G-CA treatment may increase the mRNA levels of *CYP7A1*.

## 3. Discussion

NAFLD is the most common chronic liver disease and is a rapidly growing health problem [26]. Control of these metabolic diseases is needed for a healthy life. Although several drugs to treat NAFLD are currently available, satisfactory outcomes have not been achieved. Therefore, natural products have been considered as alternative treatments to prevent NAFLD or stop its progression via several mechanisms, such as down-regulating pro-inflammatory cytokines, antioxidant effects, or by anti-dyslipidemic properties [27,28,29]. In this study, we clearly demonstrated that G-CA extract improved hypertrophy of the liver and adipose tissue, hepatic steatosis, and hyperlipidemia, and protected against liver injury in the HFD-fed NAFLD mouse model. Obesity causes hypertrophy of adipose tissue that leads to the release of diverse inflammatory cytokines, such as TNF-α and IL-1β, and these increased cytokines lead to insulin resistance through the inhibition of the insulin receptor signaling pathway [30]. Insulin resistance accelerates hepatic lipid accumulation and liver injury [31]. We observed an anti-obesity and anti-hypertrophic effect of the G-CA extract treatment in HFD-fed mice as reflected by a significant decrease in body weight gain, total fat mass, and peripheral fat pad weight. In addition, non-invasive *in vivo* quantification of fat mass measured by MRI showed that G-CA extract treatment decreased visceral and subcutaneous fat mass without any change in muscle mass.

Serum analysis showed that total cholesterol and LDL-cholesterol levels were significantly reduced in G-CA-HFD mice compared with PBS-HFD mice. Serum HDL-cholesterol levels were increased as a result of the HFD and decreased by G-CA treatment. This phenomenon may be accompanied by an increase of total cholesterol during a prolonged HFD, and others have also found that HDL-cholesterol levels were increased in the serum of a HFD induced obese mouse [15,32,33].

The liver plays an important role in lipid metabolism processes such as lipogenesis, fatty acid oxidation, and cholesterol metabolism, thus dysregulation of liver functions is a cause of metabolic disorders such as NAFLD, diabetes, and cardiovascular disease. NAFLD is characterized by excessive TG accumulation in the liver and progresses to NASH and hepatocellular carcinoma. In NAFLD pathogenesis, a “two-hit” theory, involving hepatic steatosis as a first hit and liver damage as a second hit, has been accepted [34]. The inflammatory response and oxidative stress are considered typical second hit factors [35]. In our study, the HFD increased TG accumulation in the liver; however, G-CA treatment lowered TG content. MRS is used to examine the details of the biochemical parameters and physiology as well as morphological information in metabolic disorders. We also observed a reduction of liver fat content in MRS data in the G-CA-HFD mice. However, further studies will be needed to know the changes of hepatic fat composition in the future. To understand how G-CA extract treatment decreases hepatic TG content and ameliorates liver injury and lipid metabolism, we first measured mRNA levels of inflammatory cytokines. We found that mRNA expression levels of TNF-α, IL-1β, and MCP-1 in the liver were increased in HFD-induced NAFLD mice, but were decreased in the G-CA-HFD NAFLD mice. The anti-inflammatory effect of G-CA extract may contribute to the reduction of fat accumulation in the liver and inhibition of the progress to NASH and cirrhosis.

In our study, the HFD feeding increased lipogenic gene expression in the liver, whereas G-CA extract treatment lowered SREBP-1c and FAS mRNA expression. These results suggest that reduction of hepatic steatosis by G-CA treatment was associated with a decrease in hepatic lipid synthesis. Activation of genes involved in fatty acid oxidation ameliorates hepatic lipid accumulation [36]. We therefore measured mRNA expression levels of the fatty acid oxidation related genes, sirtuin 1 (SIRT1), peroxisome proliferator-activated receptor alpha (PPARα) and carnitine palmitoyltransferase 1 alpha (CPT1A). SIRT1 plays crucial roles in fatty acid β-oxidation and cholesterol homeostasis through PPARα/peroxisome proliferator-activated receptor gamma coactivator 1 alpha (PGC-1α) [15]. The mRNA levels of SIRT1 were significantly increased in the G-CA-HFD group compared with the PBS-HFD group (Figure S2). However, there was no difference in the expression of PPARα and CPT1A between the PBS-HFD group and the G-CA-HFD group (Figure S2). It was reported that hepatic SIRT1 expression levels were decreased in the mouse and rat with NAFLD compared with non-obese subjects [15,37]. However, in our study, mRNA levels of SIRT1 were not changed in HFD-mice compared with NC-mice. Although we could not provide any direct reason for this discrepancy, one possibility is that the different duration of HFD feeding or dietary lipid composition might affect the results.

Several experimental and clinical studies have indicated that accumulation of free cholesterol and cholesterol homeostasis imbalance are relevant to the pathogenesis of NAFLD [38]. CYP7A1 is the first and the rate-limiting enzyme in bile acid synthesis, which contributes to the regulation of cholesterol metabolism. It was reported that overexpression of the CPY7A1 gene in the liver prevents HFD-induced fatty liver and decreases TG synthesis [39,40]. We found that mRNA expression levels of CPY7A1 significantly increased in the G-CA-HFD mice compared to that in NC and PBS-HFD mice. LXRα heterodimerizes with the retinoid X receptor (RXR) and binds to the LXR response element in the promoter area of CPY7A1 [41]. Our results showed that G-CA extract treatment did not affect the mRNA expression of LXRα. The FXR/RXR heterodimer binds to the FXR response element in the SHP promoter, leading to elevation of SHP expression. SHP then interacts with liver receptor homolog 1 and down-regulates CYP7A1 transcription [25]. G-CA extract treatment reduced FXR and SHP mRNA levels. Therefore, G-CA extract decreases the expression of FXR and SHP and in turn, may increase CYP7A1 mRNA expression, contributing to increased bile acid synthesis and decreased TG accumulation.

Phlorotannins, known as seaweed polyphenols, are main constituent of CA. These polyphenols are oligomers and polymers of phloroglucinol (1,3,5-trihydroxybenzen), whereas polyphenols of terrestrial plants are polymers based on flavonoids or Gallic acid [42]. Although seaweed and terrestrial polyphenols are structurally different, both polyphenols have anti-diabetic and anti-obesity effects [15]. According to our previous reports, G-CA contains phloroglucinol, eckol, phlorofucofuroeckol A, 8,8′-bieckol, dieckol, triphlorothol A and eckstolonol as major components [19]. Especially phloroglucinol contents of G-CA were higher than those of CA produced from Jeju. Further investigations are needed to clarify *in vivo* effect of phlorotannins or phloroglucinol in NAFLD animal models.

## 4. Experimental Section

### 4.1. Preparation of EtOAc Fraction of E. Cava Crude Extract

The detailed method of preparation of the EtOAc fraction of *E. cava* crude extract has been previous described [19,43]. Briefly, air-dried *E. cava* (produced in Gijang, Korea) were ground into a powder and then extracted repeatedly with MeOH for 3 h under reflux conditions. The crude extract was partitioned between CH_2_Cl_2_ and H_2_O. The organic layer was evaporated and re-partitioned between *n*-hexane and 85% aqueous MeOH. The aqueous layer was re-partitioned between *n*-BuOH and H_2_O and then the *n*-BuOH layer was fractionated with EtOAc and H_2_O. The EtOAc fraction was used for the experiments in HFD-induced obese mouse model.

### 4.2. Animals

C57BL6 mice were obtained from Orient Bio (KyeongGi-do, Seongnam, Korea). Mice were maintained under specific pathogen-free conditions in a temperature-controlled room (23 ± 1 °C) in a 12 h light/dark cycle with *ad libitum* access to food and water at the Animal Care Center, Lee Gil Ya Cancer and Diabetes Institute, Gachon University, Korea. All animal experiments were approved by the Institutional Animal Care and Use Committee of the Lee Gil Ya Cancer and Diabetes Institute.

### 4.3. Induction of Fatty Liver and Treatment with CA Extract

At 6 weeks of age, male mice were provided with either a HFD (60% fat primarily from lard, Research Diets, Inc., New Brunswick, NJ, USA, # D12492 or normal chow (5.4% fat). After 6 weeks, mice were randomly divided into three groups: the normal chow group (NC; *n* = 5), the phosphate-buffered saline (PBS)-treated HFD group (PBS-HFD; *n* = 9), and the G-CA-treated HFD group (G-CA-HFD; *n* = 7). G-CA (300 mg/kg in PBS) was administered by oral intubation daily for 10 weeks. The PBS-HFD group was administered the same volume of PBS by oral intubation. Body weight and food consumption were measured weekly. At the end of 10 weeks of treatment, the animals were sacrificed and tissues were removed for various experiments (Figure 1A).

### 4.4. Measurement of Body Composition

Fat and lean mass were determined using a ^1^H minispec system (Bruker, Karlsruhe, Germany) at 10 weeks of treatment. This equipment allowed us to analyze the weight of body fat without sedating the mice. After 10 weeks of G-CA extract treatment, the fat pads (subcutaneous and epididymal) and liver were collected, and the weights were measured.

### 4.5. MRI and MRS

MR data were acquired using an I.D. 7.2 cm volume coil on a 9.4 T Bruker MR scanner. Transverse multi-slice spin-echo images were acquired for the whole body of each mouse. Two sets of images, with fat suppression and without fat suppression, were obtained for all images. Acquisition parameters were: repetition time (TR) = 1500 ms, echo time (TE) = 20 ms, slice thickness/inter-slice distance = 1.2/1.5 mm, and field-of-view = 4.2 × 4.2 cm^2^ with a matrix size of 342 × 192. To minimize motion artifacts, respiratory gating was employed for the acquisition of abdominal area images. The whole body of each mouse was fully covered with approximately 50 slices; MRI of each fat-suppressed and non-fat suppressed slice is shown in Figure 1. All data were processed and quantified with the SliceOmatic program (Tomovision, Magog, QC, Canada). In brief, each slice was segmented into subcutaneous and visceral fat, muscle, and other tissues by utilizing a region-growing scheme based on thresholding and then all slices of the mouse were summed to yield the total amount of visceral fat, subcutaneous fat, muscle, and liver volume for each mouse. When supplementary confirmation was needed for identifying the borderline between regions, fat-suppressed MRIs were also utilized for accurate segmentation.

In order to quantify hepatic lipids *in vivo*, water spectra using a stimulated echo acquisition mode (STEAM) sequence were obtained, and the water signal was utilized as the internal reference for quantification. MRS, both with and without water suppression, was assessed in the liver with voxel size of 2.5 × 2.5 × 2.5 mm^3^. TR/TE was 6000/3 msec for all STEAM MRS. ^1^H MRS for liver fat content were processed using NUTS (Acorn NMR, Livermore, CA, USA), liver fat percentage (%) was calculated by the methylene peak at 1.3 ppm in water suppressed MRS relative to the water peak at 4.7 ppm in water MRS as previously described [44,45].

### 4.6. Serum Analysis

After 10 weeks of G-CA extract treatment, blood samples were collected from the orbital sinus under anesthesia after 3 h of food deprivation. Blood samples were centrifuged at 3000 *g* for 20 min, and serum levels of alanine aminotransferase (ALT), aspartate aminotransferase (AST), cholesterol, triglycerides (TG), low-density lipoprotein (LDL)-cholesterol, and high-density lipoprotein (HDL)-cholesterol were measured using a Beckman Coulter AU 480 (Beckman Coulter, Pasadena, CA, USA). The atherogenic index was calculated by using the formula = ([TC] − [HDL-C])/[HDL-C].

### 4.7. Analysis of mRNA Expression by Quantitative Real-Time PCR

Total RNA was isolated from liver tissue using the TRIZOL reagent (Invitrogen, Carlsbad, CA, USA) following the manufacturer’s instructions, and cDNA was synthesized using a PrimeScript 1st strand cDNA synthesis kit (Takara Bio, Shiga, Japan). Quantitative real-time PCR was performed using the SYBR Premix Ex Taq (Takara Bio, Shiga, Japan) and Bio-Rad CFX384 Touch Real-time PCR detection system (Bio-Rad, Hercules, CA, USA). PCR was carried out and stopped at 40 cycles (2 min at 50 °C, 10 min at 95 °C, and 40 cycles of 10 s at 95 °C and 1 min at 60 °C). The primer sequences used are shown in Table 1. Relative copy number was calculated using the threshold crossing point (*C*_t_) as calculated by the ΔΔ*C*_t_ calculations.

**Table 1:** Primer sequences of mouse mRNA.

Target	Forward Primer	Reverse Primer
TNFα	CCAACGGCATGGATCTCAAAGACA	AGATAGCAAATCGGCTGACGGTGT
IL-1β	CTACAGGCTCCGAGATGAACAAC	TCCATTGAGGTGGAGAGCTTTC
MCP-1	TTAAAAACCTGGATCGGAACCAA	GCATTAGCTTCAGATTTACGGG
SREBP1c	GGAGCCATGGATTGCACATT	GGCCCGGGAAGTCACTGT
FAS	GCTGCGGAAACTTCAGGAAAT	AGAGACGTGTCACTCCTGGACTT
ACC1	ACGCTCAGGTCACCAAAAAGAAT	GTAGGGTCCCGGCCACAT
CYP7A1	AGCAACTAAACAACCTGCCAGTACTA	GTCCGGATATTCAAAGGATGCA
FXR	TCCGGACATTCAACCATCAC	TCACTGCACATCCCAGATCTC
SHP	AGGAACCTGCCGTTCCTTCTG	TGGCTTCCTCTAGCAGGATC
HNF4α	CCAACCTCAATTCATCCAACA	CCCGGTCCGCCACAGAT
LXRα	AGGAGTGTCGACTTCGCAAA	CTCTTCTTGCCGCTTCAGTTT
18S rRNA	CCATCCAATCGGTAGTAGCG	GTAACCCGTTGAACCCCATT

### 4.8. Oil-Red O Staining

Liver pieces were embedded in optimal cutting temperature compound. Frozen liver sections were cut at 7 μm thickness and stained with Oil Red O and Mayer’s hematoxylin solution for microscopy [19].

### 4.9. Quantification of Liver Triglyceride Content

Liver TG content was measured as previously described [19]. Briefly, liver tissue (50 mg) was homogenized with ethanolic KOH (2 parts EtOH: 1 part 30% KOH) overnight and then KOH and distilled water were added to the homogenate. After centrifugation (1000 *g* for 5 min), the supernatant was mixed with 1M MgCl_2_. TG content was measured in the solution using a Cleantech TG-S kit (Asan Pharmaceutical Company, Seoul, Korea).

### 4.10. Statistical Analysis

Data are presented as mean ± SE. The significance of differences was analyzed with one-way ANOVA followed by the Newman-Keuls procedure using GraphPad Prism v5.0 (GraphPad Sofrware, Inc., La Jolla, CA, USA). The value of statistical significance was set at *p* < 0.05.

## 5. Conclusions

This study showed that the polyphenol-rich G-CA extract reduced liver volumes and liver fat accumulation in HFD-fed mice as evidenced by *in vivo* MRI and MRS evaluation. G-CA extracts inhibited inflammatory activity and improved lipid metabolism, and consequently, ameliorated hepatic steatosis. Therefore, G-CA extract might have therapeutic potential in the management of NAFLD.

## Supplementary Files

Supplementary File 1
