# Supplementary material for: Polyphenol-Rich Fraction of Ecklonia cava Improves Nonalcoholic Fatty Liver Disease in High Fat Diet-Fed Mice
Source: Mar Drugs. 2015 Nov 12;13(11):6866–83. doi: 10.3390/md13116866 (PMC4663557; doi:10.3390/md13116866)
Supplement: Supplementary File 1 [file marinedrugs-13-06866-s001.doc]

**Supplementary Information**


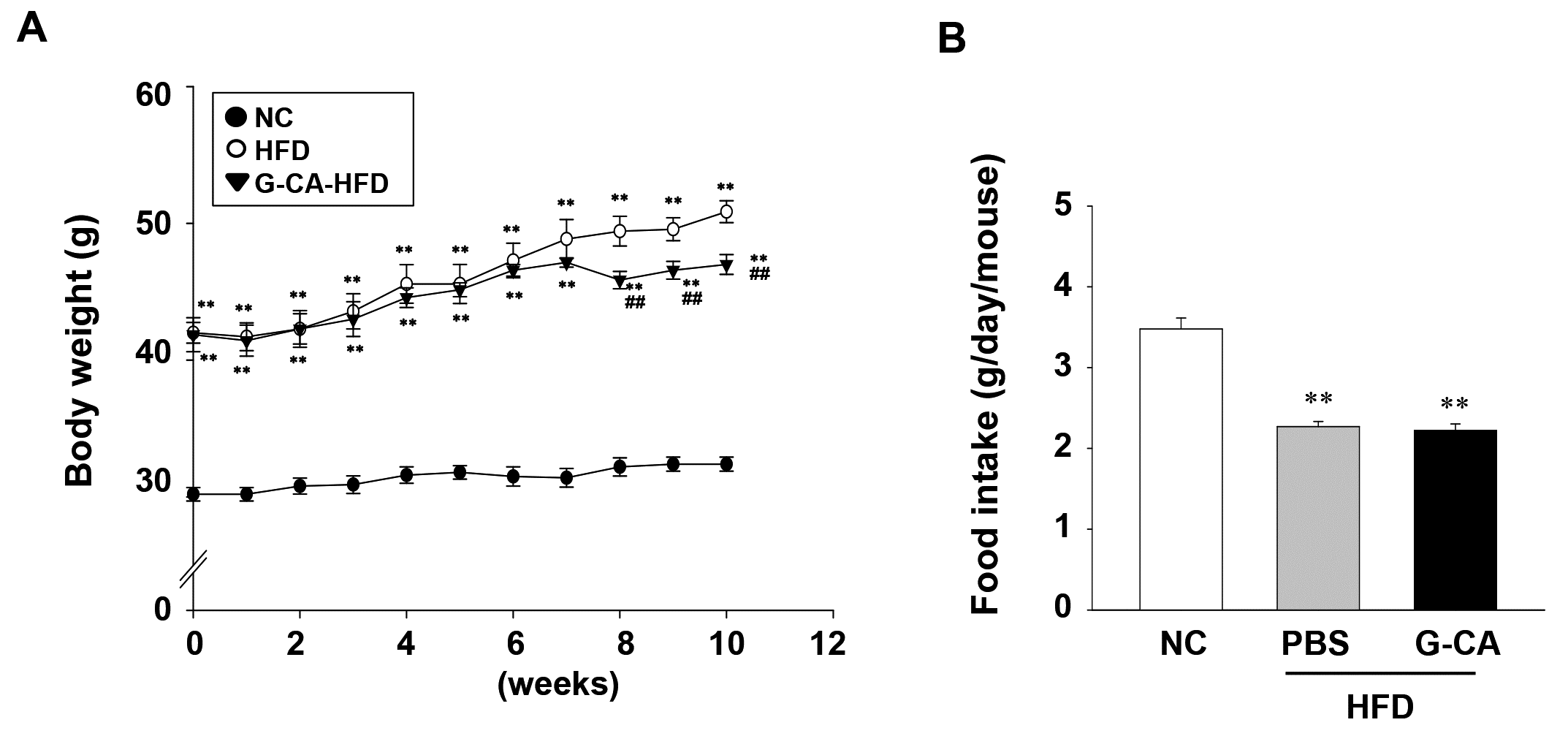


**Figure S1.** Effect of G-CA extracts on body weight and food intake. (**A**) Body weights were monitored weekly; (**B**) Food intake was measured weekly. Values are the average weight of food consumed/mouse/day. Values are mean ± SE. ** *p* <0.01 *vs.* NC group;
^##^ *p* < 0.01 *vs.* PBS-HFD group.


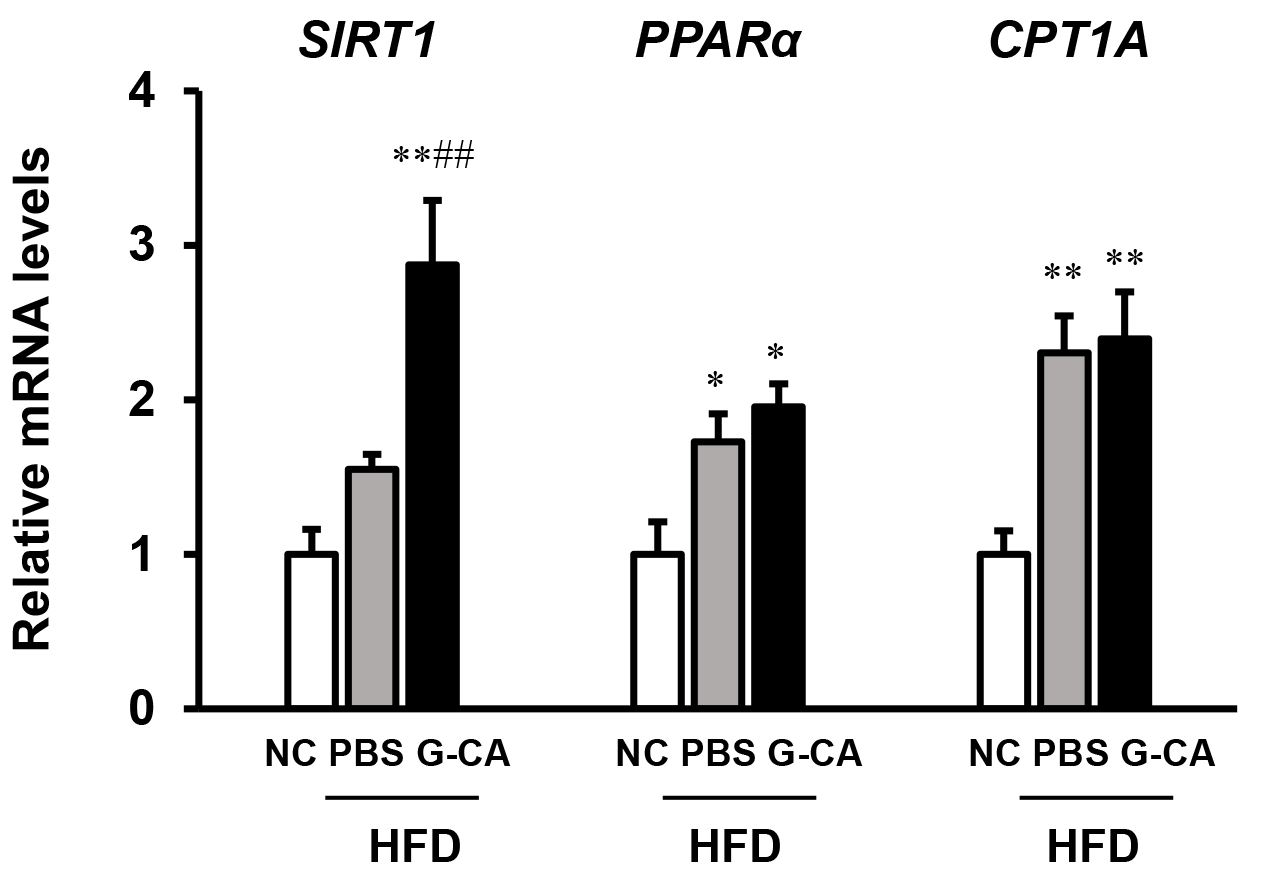


**Figure S2.** Effect of G-CA on fatty acid oxidation related gene expression in liver.
Six weeks after beginning a high fat diet, C57BL6 mice were orally administered
G-CA (300 mg/kg body weight) or PBS daily for 10 weeks. Sirtuin 1 (SIRT1), Peroxisome proliferator-activated receptor alpha (PPARα) and carnitine palmitoyltransferase 1 alpha (CPT1A) mRNA levels were analyzed by RT-qPCR and normalized to 18s rRNA expression. Values are expressed as fold change compared with the NC group. NC: untreated, normal chow diet; PBS-HFD: PBS-treated, high fat diet (HFD); G-CA-HFD:
G-CA-treated, HFD. Values are mean ± SE. * *p* <0.05, ** *p* <0.01 *vs.* NC group; ^##^ *p* < 0.01 *vs.* PBS-HFD group.
